# ERP evidence for the recognition of emotional prosody through simulated cochlear implant strategies

**DOI:** 10.1186/1471-2202-13-113

**Published:** 2012-09-20

**Authors:** Deepashri Agrawal, Lydia Timm, Filipa Campos Viola, Stefan Debener, Andreas Büchner, Reinhard Dengler, Matthias Wittfoth

**Affiliations:** 1 Department of Neurology, Hannover Medical School, Hannover, Germany; 2 Department of Psychology, Carl von Ossietzky Universität, Oldenburg, Germany; 3 Department of Otolaryngology, Hannover Medical School, Hannover, Germany

**Keywords:** Emotional prosody, Cochlear implants, Simulations, Event-related potentials

## Abstract

**Background:**

Emotionally salient information in spoken language can be provided by variations in speech melody (prosody) or by emotional semantics. Emotional prosody is essential to convey feelings through speech. In sensori-neural hearing loss, impaired speech perception can be improved by cochlear implants (CIs). Aim of this study was to investigate the performance of normal-hearing (NH) participants on the perception of emotional prosody with vocoded stimuli. Semantically neutral sentences with emotional (happy, angry and neutral) prosody were used. Sentences were manipulated to simulate two CI speech-coding strategies: the Advance Combination Encoder (ACE) and the newly developed Psychoacoustic Advanced Combination Encoder (PACE). Twenty NH adults were asked to recognize emotional prosody from ACE and PACE simulations. Performance was assessed using behavioral tests and event-related potentials (ERPs).

**Results:**

Behavioral data revealed superior performance with original stimuli compared to the simulations. For simulations, better recognition for happy and angry prosody was observed compared to the neutral. Irrespective of simulated or unsimulated stimulus type, a significantly larger P200 event-related potential was observed for happy prosody after sentence onset than the other two emotions. Further, the amplitude of P200 was significantly more positive for PACE strategy use compared to the ACE strategy.

**Conclusions:**

Results suggested P200 peak as an indicator of active differentiation and recognition of emotional prosody. Larger P200 peak amplitude for happy prosody indicated importance of fundamental frequency (F0) cues in prosody processing. Advantage of PACE over ACE highlighted a privileged role of the psychoacoustic masking model in improving prosody perception. Taken together, the study emphasizes on the importance of vocoded simulation to better understand the prosodic cues which CI users may be utilizing.

## Background

In humans, speech is the most important type of communication. Verbal communication conveys more than the syntactic and semantic content. Besides explicit verbal content, emotional non-verbal cues are a major information carrier. The term ‘prosody’ describes the non-propositional cues, including intonations, stresses, and accents [1]. The emotional speech tends to vary in terms of three important parameters. Among these, most crucial is the fundamental frequency (F0), followed by duration, and intensity [2]. A great deal of work in neuropsychology has focused on emotional prosody in normal-hearing (NH) individuals and in neurological conditions such as Parkinson’s disease [3] and primary focal Dystonia [4] but rarely in individuals with hearing loss. Individuals with severe to profound hearing loss have a limited dynamic range of frequency, temporal and intensity resolution, thus impairing their perception of prosody.

Cochlear implants (CIs) enable otherwise deaf individuals to achieve levels of speech perception that would be unattainable with conventional hearing aids [5,6]. The outcome of CI depends on many factors, such as the etiology of deafness, age of implantation, duration of use, electrode placement, and cortical reorganization [7,8]. In a CI, speech signals are encoded into electrical pulses to stimulate hearing nerve cells. Algorithms used for such encoding are known as speech-coding strategies. An important possible variability in hearing performance of CI users may reside in the speech-coding strategy used [9]. There is a need to understand the contribution of this source of variability to improve perception. NH adults perceive a variety of cues to identify information in the speech spectrum, some of which may be especially useful in the context of spectrally-degraded speech. Simulations that mimic an acoustic signal in a manner consistent with the output of a CI have been proven helpful for comprehending the mechanism of electric hearing [10], as they provide insight into the relative efficacy of different processing algorithms.

The aim of this study was to play vocoded (simulated) sentences to NH subjects to determine if speech-coding strategies are comparable on prosody perception. In the present experiment, signals vocoded with the Advance Combination Encoder (ACE) and Psychoacoustic ACE (PACE), commercially known as MP3000 were used [11,12]. Both ACE and PACE are N-of-M-type strategies, i.e., these strategies select fewer channels (N) per cycle from (M) active electrodes (N out of M). In ACE, (N of M) bands (or electrodes) with highest amplitude are stimulated in each stimulation cycle, where (M) is the number of electrodes available [13] e.g., 8–12 bands with the maximum amplitude are selected out of 22. This method of selection aims at capturing perceptually relevant features, such as the formant peaks.

The new PACE strategy [14] is an ACE variant based on a psychoacoustic masking model. This algorithm is akin to the MP3 audio-format used for transferring music. This model describes masking effects that take place in a healthy auditory system. Thus, the (N) bands that are most important for normal hearing are delivered, rather than merely the spectral maxima, as with the ACE. It can be speculated that such an approach could improve spectral resolution, thereby improving speech perception.

However, comparisons of the new PACE strategy with established ACE are scarce. In past, researchers tested PACE on sentence recognition tasks in speech-shaped noise at 15 dB signal-to-noise ratios and compared it with ACE [11]. A large improvement of PACE was found when four channels were retained, but not for eight channels. In their study, [15] the authors compared ACE and PACE on musical instrument identification and did not find any difference in terms of music perception. In another study researchers found an improvement in the Hochmair, Schulz, and Moser (HSM) sentence test score for PACE (36.7%) compared with ACE (33.4%), indicating advantage of PACE over ACE [16]. Taken together, these studies reflect mixed results, which might be due to the lack of objective dependent variables used. To overcome this issue, event-related potentials (ERPs) could be used, as they do not rely on subjective, behavioral output measures.

Previous research has shown that ERPs are important for studying normal [17] and impaired processing of emotional prosody differentiation and identification [18]. Researchers recorded visual ERPs on words with positive and negative emotional connotations and reported that the P200 wave reflects general emotional significance [19]. Similar results were reported for the auditory emotional processing [20,21]. Researchers [22] reported that with ERPs, emotional sentences can be differentiated from each other as early as 200 ms after sentence onset, independent of speaker voices. Although in the aforementioned studies the auditory N100 has not been focused on, it is believed to reflect perceptual processing and is modulated by attention [23,24].

The present study aimed to elucidate differences between the effects of the ACE and PACE coding strategies on emotional prosody recognition. We hypothesized that, regarding the identification of verbal emotions, PACE may outperform ACE, which should be reflected in behavioral measures and auditory ERPs.

## Results

### Behavioral results

#### Reaction time

Mean RTs for each emotional condition for both subject groups are listed in Table 1. These response times were corrected for sentence length by subtracting this variable from each individual response. Note that RTs calculated here were post-stimulus offset RTs. The ANOVA revealed a significant main effect of factor emotional prosody, *F*(2, 38) = 30.102, *p* < .001. Further, the main effect of stimulus type, strategy and interaction of factors were not significant. To understand the main effect of emotional prosody, follow up analysis was then performed. Reaction times were significantly shorter for happy, *t* (39) = 6.970, *p* =.011, and for angry, *t* (39) = 7.301, *p* = .001, than neutral. But there was no difference between happy and angry. Overall, it was demonstrated that, subjects were faster to respond to sentences with happy and angry prosodies compared with neutral.

**Table 1:** Mean reaction time and accuracy rates with standard deviations in parenthesis for all three emotions

**Conditions**	**Neutral**	**Angry**	**Happy**
Reaction time (seconds)			
Original (unsimulated)	0.66 (0.23)	0.48 (0.25)	0.48 (0.22)
ACE simulations	0.65 (0.20)	0.50 (0.20)	0.53 (0.20)
PACE simulations	0.68 (0.20)	0.50 (0.20)	0.55 (0.22)
Accuracy rate (%)			
Original (unsimulated)	97% (5.0)	97% (5.0)	97% (5.0)
ACE simulations	77% (22.0)	82% (13.0)	70% (17.0)
PACE simulations	85% (17.0)	88% (13.0)	86% (15.0)

### Accuracy rate

In order to investigate whether happy and angry prosodies would be recognized more easily than neutral prosody, accuracy rates were compared for all sentences. In general, emotional prosody detection was above chance level (50%) for both unsimulated and simulated sentences. Computed for all emotions together, subjects achieved an average of 97% accuracy for unsimulated and 80% for simulated sentences. On ANOVA, significant main effect of stimulus type was observed, *F*(1, 18) = 32.442, *p* = .001. The results indicated that, irrespective of emotional prosody, unsimulated sentences produced higher identification rates than simulated. Further, the significant main effect of strategy was observed, *F*(1, 18) = 4.825, *p* = .038. This indicated that participants perceiving PACE simulations were more accurate in emotional prosody identification compared to those with ACE. In addition, interaction between stimulus type and strategy was significant, *F*(1, 18) = 4.982, *p* = .039. Follow up t-tests revealed that accuracy scores with simulated PACE were higher than simulated ACE, *t* (9) = 3.973, *p* = .003, for happy but not for neutral and angry prosody. However, unsimulated PACE and unsimulated ACE did not show significant differences on accuracy of recognition. The accuracy rates for emotional prosody identification are depicted in Table 1. All other effects and interactions did not reach significance.

## ERP results

**Figure 1:**
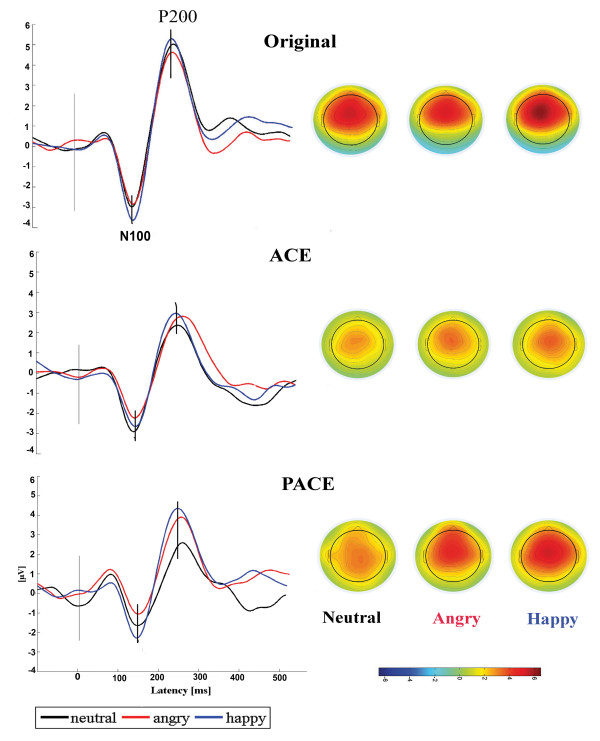
**ERP waveforms for three emotional prosodies for simulated and unsimulated conditions.** Average ERP waveforms recorded at the Cz electrode in original (unsimulated) and simulated conditions for all three emotional [neutral (black), angry (red) and happy (blue)] stimuli from 100 ms before onset to 500 ms after the onset of the sentences with respective scalp topographies at P200 peak (X-axis: latency in milliseconds, Y-axis: amplitude in μV). Top: N100-P200 waveform for original sentences. Middle: waveform for ACE simulations, and Bottom: waveform for PACE simulations.

An N100-P200 complex, shown in Figure 1, characterized the ERP waveforms elicited after sentence onset in the present experiment.

### N100

The main effect of emotional prosody on the N100 latency measure did not reach significance. No significant main effect of factor stimulus type or strategy observed. Similarly, the interactions between factors were not significant.

For the analysis of N100 amplitude, ANOVA revealed main effects of emotional prosody, *F*(2, 38) = 7.902, *p* = .001, and strategy, *F*(1, 18) = 5.634, *p* = .029, indicating significant differences between the strategies. The interaction between emotional prosody and strategy was also significant, *F*(2, 38) = 3.951, *p* = 029. Follow up paired *t*-test revealed that the N100 amplitude for ACE strategy was significantly more negative for angry emotion, *t* (9) = 2.803, *p* = .021, compared with PACE. The N100 peak amplitude for happy and neutral emotion, did not differ between ACE and PACE. The latency and amplitude are displayed in Table 2, with standard deviations shown in parentheses.

**Table 2:** Mean N100 latency in milliseconds and amplitude in micro-volts with standard deviation for all emotions

**Conditions**	**Neutral**	**Angry**	**Happy**
Latency (ms)			
Original (unsimulated)	137 (11.5)	138 (13.5)	140 (9.0)
ACE simulations	132 (20.0)	140 (15.8)	134 (17.2)
PACE simulations	140 (15.8)	148 (13.3)	148 (15.5)
Amplitude (μV)			
Original (unsimulated)	−3.90 (1.8)	−3.90 (1.5)	−4.0 (1.9)
ACE simulations	−3.90 (1.9)	−3.67 (1.6)	−3.80 (1.8)
PACE simulations	−3.80 (1.5)	–3.0 (1.2)	−3.70 (1.3)

### P200

With respect to P200 latency, the factor emotional prosody displayed significant main effect, *F*(2, 38) = 4.882, *p* = .013. Further, analysis revealed significant main effect of stimulus type, *F*(1, 18) =4.84, *p* = .040, such that the latency of P200 peak was delayed for simulated sentences compared to unsimulated sentences. Follow up paired t-tests revealed that P200 latency was delayed for simulated happy prosody compared to simulated angry prosody, *t* (19) = 2.417*, p* = .026. No other main effects, interactions or pair-wise comparisons reach significance.

With respect to the amplitude analysis, the ANOVA revealed a significant main effect of emotional prosody indicating waveform differences between emotional sentences, *F*(2,38) = 5.982, *p* = .006. Statistical values for the emotional effects of these comparisons are as follows: (i) happy vs. angry, *t* (39) = 2.117*, p* = .036 (ii) happy vs. neutral, *t* (39) = 2.943*, p* = .006. Results also revealed a main effect of stimulus type, *F*(1, 18) = 13.44, *p* = .002, indicating significantly reduced peak amplitude for simulated compared with unsimulated sentences. This effect was significant for all three emotions. There was no main effect of factor strategy observed. However, a significant interaction between emotional prosody and strategy, *F*(2, 38) = 3.934, *p* = .029, was seen. The amplitude evoked by happy prosody was significantly larger compared with neutral, *t* (9) = 2.424, *p* = .038, and compared with angry, *t* (9) = 4.484, *p* = .002, for PACE users. In addition, a significant 3-way interaction between emotional prosody x stimulus type x strategy, *F*(2, 38) = 4.302, *p* = .021 was observed. Follow up results revealed that for unsimulated condition there was no difference between ACE and PACE. The factor emotional prosody also showed no significant effect. However, for simulated condition, amplitude differences were evident between ACE and PACE on emotional prosody. It was observed that amplitude of P200 for happy prosody was significantly larger with simulated PACE compared to simulated ACE, *t* (9) = 3.528, *p* = .007. The amplitude of P200 for neutral and angry prosody did not significantly differ between simulated ACE and PACE. No other pair wise comparisons showed significant differences. The latency and amplitude are displayed in Table 3, with standard deviations shown in parentheses.

**Table 3:** Mean P200 latency in milliseconds and amplitude in micro-volts with standard deviation for all emotions

**Conditions**	**Neutral**	**Angry**	**Happy**
Latency (ms)			
Original (unsimulated)	240 (16.6)	240 (20.0)	234 (16.0)
ACE simulations	244 (26.1)	242 (30.6)	242.4 (21.2)
PACE simulations	246 (13.6)	248 (21.6)	254.8 (20.0)
Amplitude (μV)			
Original (unsimulated)	5.9 (1.5)	6.0 (1.5)	6.2 (1.8)
ACE simulations	3.6 (1.5)	4.2 (1.3)	4.2 (0.9)
PACE simulations	3.6 (1.4)	5.2 (1.4)	5.6 (1.5)

Taken together, the results demonstrated a significant difference in emotional prosody identification. In all comparisons the happy prosody elicited stronger P200 amplitudes than other two emotional prosodies. In addition, the interactions were significant, suggesting that each simulation type had different effects on emotion recognition.

## Discussion

This study aimed to investigate an early differentiation of vocal emotions in semantically neutral expressions. By utilizing behavioral tasks and ERPs to investigate neutral, angry, and happy emotion recognition, we demonstrated that performance of normal hearing subjects were significantly better for unsimulated than for CI-simulated prosody recognition. Similarly the performance with PACE was better compared to ACE.

For post-offset RTs, participants were faster to identify happy and angry prosodies compared with the neutral emotion. These findings are in parallel with findings in literature on prosody processing that have constantly shown the faster recognition of emotional stimuli compared with neutral stimuli [25-28]. The aforementioned studies have attributed this rapid detection of vocal emotions to the salience and survival value of emotions over neutral prosody. Moreover, an emotional judgment of prosody might be performed faster, as non-ambiguous emotional associations are readily available. In contrast, neutral stimuli may elicit positive or negative associations which otherwise may not exist. Thus, the reaction times may simply reflect a longer decision time for neutral compared with emotional sentences.

For the accuracy rate analysis, near perfect scores (97% correct) were obtained when participants heard original unsimulated sentences. These findings are higher than the results (90 to 95%) reported in previous studies [29,30]. This substantiates that the speaker used in the current study accurately conveyed the three target emotions. Thus, the stimuli bank used in the present experiment appears to be appropriate for conveying the requisite prosodic features needed to investigate different CI strategies on the grounds of emotion recognition.

The ERP data for emotional prosody perception recorded in all the participants demonstrated differential electrophysiological responses in the sensory-perceptual component of emotion relative to neutral prosody. The auditory N100 component is a marker of physical characteristics of stimuli such as temporal pitch extraction [31]. Evidence exists in the literature advocating the N100 as the first stage of emotional prosody processing [32]. In the current study, N100 amplitude was more negative for ACE strategy use suggesting early stages of prosody recognition might be adversely affected by stimulus characteristics. However, N100 is modulated by innumerable factors including attention, motivation, arousal, fatigue, complexity of the stimuli, and methods of recording etc. [33]. Thus, it is not possible to delineate the reasons for presence of the N100 as one cannot rule out the contribution of above mentioned factors to the observed results. The next stage of auditory ERP processing is the P200 component.

The functional significance of the auditory P200 component has been suggested to index stimulus classification [34] but the peak P200 is also sensitive to different acoustic features such as pitch [35], intensity [36] and duration. For instance, in studies of timbre processing, P200 peak amplitudes were found to increase with the number of frequencies present in instrumental tones [37,38]. The emotional prosody processing occurring around 200 ms reflects the integration of acoustic cues. These cues help participants to deduce emotional significance from the auditory stimuli [32]. A series of experiments [22,39,40] have enunciated that the P200 component is modulated by spectral characteristics and affective lexical information.

In the present study, it was evident that the P200 peak amplitude was largest for the happy prosody compared with the other two. These results are in line with previous reports [41] where ERPs were recorded as participants judged the prosodies. It was seen that the P200 peak amplitude was more positive for the happy prosody, suggesting enhanced processing of positive valence. In an imaging study, researchers found that activation in the right anterior and posterior middle temporal gyrus, and in the inferior frontal gyrus, was larger for happy intonations compared with angry intonations [42]. This enhanced activation was interpreted as highlighting the role of happy intonation as socially salient cues involved in the perception and generation of emotional responses when individuals attend to the voices. In a study measuring ERPs, Spreckelmeyer and colleagues reported a larger P200 component amplitude for happy voice compared with sad voice tones [43]. They attributed these results to the spectral complexity of happy tones, including F0 variation, as well as sharp attack time. In our study the acoustical analysis of the stimuli also revealed higher mean F0 values, and wider ranges of F0 variation for the happy prosody compared with the angry and neutral prosodies. These F0-related parameters of the acoustic signal may thus serve as early cues for emotional significance and accordingly may facilitate task-specific early sensory processing. These results are well in line with earlier work [2] confirming pitch cues as the most important acoustical dimension in emotion recognition. The fact that the happy prosody recognition elicited larger P200 peak amplitude, even on simulation, signifies the robustness of F0 parameters that are well preserved, even after the degradation of speech. There is evidence from an ERP study to suggest that negative stimuli are less expected and take more effort to process compared with positive stimuli [44]. Thus, the larger F0 variation, as well as lower intensity variation, early in the spectrum of the happy prosody and the social salience could have resulted in improved happy prosody recognition.

Auxiliary to the aim of affective prosody recognition in unsimulated vs. simulated sentences, the study intended to throw light on differences between two types of CI strategies. Irrespective of the type of strategy simulated, all subjects performed above chance level on simulations. It was seen that the performance of subjects for simulations was poorer than unsimulated sentences for all emotions. This could be attributed to a very limited dynamic range that was maintained while creating the simulations to mimic the real implants as much as possible. Secondly, the algorithms used to create simulations degrade the spectral and temporal characteristics of the original signal. As a result, access to several F0 cues essential for emotion differentiation, is not available to the same extent as in the unsimulated situation [45]. Although the vocoders used to create simulations adulterate the stimuli, they are still the most analogous to imperfect real-life conditions such as perception through cochlear implants [46].

The final aspiration of this study was to compare the speech-coding strategies and find out which one is better for prosody recognition. From the results of the comparison of prosody perception with two simulation strategies, i.e. PACE and the ACE, the results indicated noticeable advantages of PACE over the currently popular ACE strategy, and the difference was most evident for the happy emotion. The larger P200 component effect for happy prosody was observed for PACE compared with ACE simulations. This larger amplitude seen for PACE may be attributed to its coding principle that result in a greater dispersion and less clustering of the channels stimulated. Past experiments reported that speech perception is better for subjects using PACE compared with the ACE strategy. Similarly, [47] predicted that PACE might have an advantage over the ACE in music perception. Although both ACE and PACE are N of M strategies, coding in the PACE strategy is a result of a psychoacoustic masking model. The bands selected by this model are based on the physiology of normal hearing cochlea. This model extracts the most meaningful components of audio signals and discards signal components that are masked by other noisy components and are, therefore, inaudible to normal hearing listeners. Due to this phenomenon, the stimulation patterns inside the cochlea are more natural with the PACE [11], meaning that the presented stimuli sounds more natural and less stochastic. As the ACE strategy lacks such a model, a stimulation pattern similar to normal hearing cochlea can never be created, resulting in unnatural perception due to undesirable masking effects in the inner ear. This explains the poor performance on both the behavior and ERPs when ACE simulations were heard. Additionally other reason for this further improvement could be that, unlike for ACE, the bands selected by the masking model are widely distributed across the frequency range in PACE. This decreases the amount of electric field interaction, leading to an improvement in speech intelligibility by preserving important pitch cues. Thus, in PACE only the most perceptually salient components, rather than the largest components of the stimulus, are delivered to the implant, preserving the finer acoustic features that otherwise would have been masked leading to improved spectral and temporal resolution, thereby enhancing verbal identification and differentiation compared with ACE.

## Conclusions

In accordance with a previous report [22], the present study proposes that it is possible to differentiate emotional prosody as early as 200 ms after the sentence onset, even when sentences are acoustically degraded. Acoustic analyses of our study, as well as studies carried out previously, indicated that the mean pitch values, the ranges of pitch variation and overall amplitudes are strong acoustic indicators for the targeted vocal emotions. Secondly, our results suggest that PACE is superior to ACE in regard to emotional prosody recognition. The present study also confirms that simulations are useful for comparing speech coding strategies as they mimic the limited spectral resolution and unresolved harmonics of speech processing strategies. However, as pointed out by [46], results of simulation studies should be interpreted with caution as vocoders may have significant effects on temporal and spectral cues. Thus, emotional prosody processing in CI users awaits further research. Future implant devices and their speech processing strategies will increase the functional spectral resolution and enhance the perception of salient voice pitch cues to improve CI users’ vocal emotion recognition. The implementation of the psychoacoustic masking model that went into the development of PACE seems an important step towards achieving this goal.

## Methods

### Participants

The group of participants consisted of twenty right-handed normal-hearing native German speakers with a mean age of 41 years (range: 25–55 years, SD = 7.1). Subjects were randomly divided into two subgroups. The first group (Group I) consisted of ten individuals with a mean age of 40 years (SD = 8.1) presented with an ACE simulation perception task. The second group (Group II) comprised ten subjects with a mean age of 42 years (SD = 6.3) performing a PACE simulation task. Subjects had no history of neurological, psychiatric or hearing illness or speech problems. Application of the Beck's Depression Inventory (BDI) revealed that none of the subjects scored higher than nine points that suggested no significant depressive symptoms present. The study was carried out in accordance with the Declaration of Helsinki principles and was approved by the Ethics Committee of the Hannover Medical School. All participants gave written consent prior to the recording and received monetary compensation for their participation.

### Stimuli

**Figure 2:**
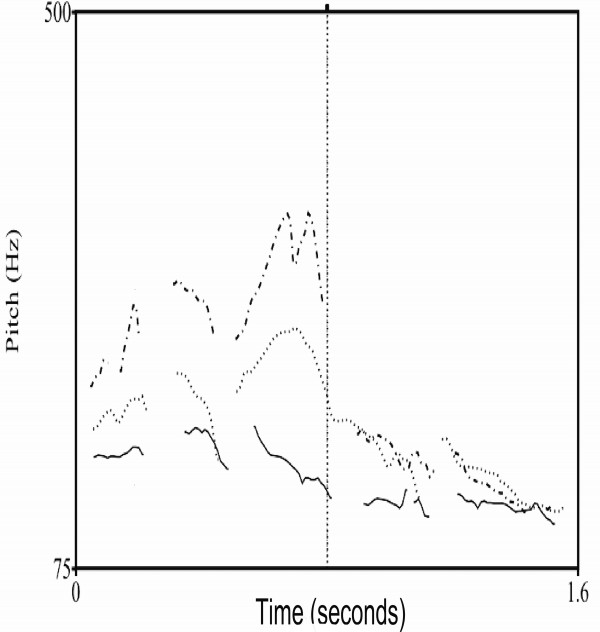
**Pitch contours of the three emotions.** The Praat generated pitch contours of neutral (solid line), angry (dotted line) and happy prosody (dashed line) for the original (unsimulated) sentence: “Sie hat die Zeitung gelesen”.

**Figure 3:**
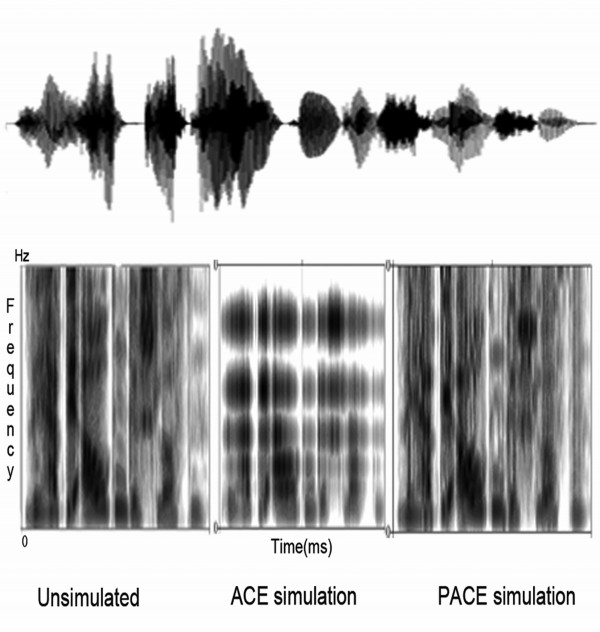
**Spectrograms of the simulated and unsimulated stimuli.** Spectrograms (as deduced by Praat software) of three stimuli type for a happy sentence. Top: visible sound of the happy sentence. Bottom: spectrograms of the same sentence. Left: Original (unsimulated) sentence. Centre: ACE simulation and Right: PACE simulation.

Fifty semantically neutral sentences spoken by a professional German actress served as the stimulus material for the experiment. Each sentence was spoken with three different emotional non-verbal cues, resulting in fifty stimuli for each emotion (neutral, happy and angry). In total 150 sentences were used for the experiment. Every stimulus was taped with a digital audio tape recorder with a sampling rate of 44.1 kHz and digitized at 16-bit [20]. These sentences are from the stimuli bank that several researchers have used previously, e.g., [20] used above sentences to study the lateralization of emotional speech using fMRI. Similarly, [48] studied valence-specific differences of emotional conflict processing with these sentences. All sentences had the same structure (e.g., “*Sie hat die Zeitung gelesen*”; “She has read the newspaper”). To create simulations of these natural sentences mimicking the ACE and PACE strategies, the Nucleus Implant Communicator (NIC) Matlab toolbox was used [49]. All stimuli were acoustically analyzed using Praat 5.1.19 to gauge the acoustic differences between emotions [50]. Differences in the fundamental frequency (F0), overall pitch (see Figure 2), intesity and duration of the sentences were extracted. Values for the acoustic features from sentence onset to sentence offset are presented in Table 4. Figure 3 illustrates the spectrogram for unsimulated, ACE-simulated and PACE-simulated sentences.

**Table 4:** Acoustic parameters of unsimulated and simulated sentences (standard deviations in parenthesis) for all emotions

**Strategy**	**Stimulus**	**Mean duration (secs)**	**Mean F0 (Hz)**	**Mean intensity (dB)**
Original (Unsimulated)	Neutral	1.60 (0.3)	157.0 (23.0)	68.6 (1.0)
	Angry	1.70 (0.3)	191.5 (25.0)	70.0 (0.9)
	Happy	1.80 (0.4)	226.6 (24.6)	67.3 (0.9)
ACE	Neutral	1.68 (0.2)	130.1 (28.8)	75.2 (1.0)
	Angry	1.75 (0.2)	117.9 (29.0)	77.7 (0.9)
	Happy	1.81 (0.24)	123.2 (33.0)	76.1 (1.3)
PACE	Neutral	1.68 (0.2)	161.0 (28.9)	72.0 (0.9)
	Angry	1.75 (0.2)	189.7 (25.6)	75.5 (0.9)
	Happy	1.88 (0.23)	222.0 (32.3)	73.7 (1.3)

### Procedure

The experiment was carried out in a sound-treated chamber. Subjects were seated in a comfortable armchair facing a computer monitor, placed at a distance of one meter. Stimuli were presented with the ‘Presentation’ software (Neurobehavioral system, version 14.1) in a random order via loudspeakers positioned to the left and right of the monitor at a sound level indicated by participants to be sufficiently audible. All stimuli were randomized in such a way that the same sentence with two different emotions did not occur in succession. Stimuli were presented at a fixed presentation rate with an inter-trial-interval of 2500 ms. Participants were instructed to identify as accurately as possible whether the sentence had a neutral, happy or angry prosody and then press the respective response key as a marker of their decision after the end of a sentence. Each key on a response box corresponded to one of three prosodies. The matching of buttons to responses was counterbalanced across subjects within each response group. The experiment consisted of one randomized unsimulated run and one randomized simulated run of approximately thirteen minutes each. The blocks of unsimulated and simulated sentences were counterbalanced across participants. Only the responses given after the completion of a sentence were included in later analyses. Accuracy scores and reaction times were calculated for each emotion for unsimulated and simulated sentence and were subjected to SPSS (10.1) for statistical analysis.

### ERP procedure

Continuous Electroencephalography (EEG) recordings were acquired using a 32-channel BrainAmp (BrainProducts, Germany, http://www.brainproducts.de) EEG amplifier. An active electrodes embedded cap (BrainProducts, Germany, http://www.brainproducts.de) with thirty Ag/Ag-Cl electrodes was placed on the scalp according to the International 10–20 system [51], with the reference electrode on the tip of the nose. Vertical and lateral eye movements were recorded using two electrodes, one placed at the outer canthus and one below the right eye of the participants. Impedances of the electrodes were kept below 10KΩ. The EEG was recorded continuously on-line and stored for off-line processing. The EEGLAB [52] open source software version (9.0.4.5s) that runs under the MATLAB environment was used for analysis. The data were band-pass filtered (1 to 35 Hz) and trials with non-stereotypical artifacts that exceeded inbuilt probability function (jointprob.m) by three standard deviations were removed. Independent component analysis (ICA) was performed with the Infomax ICA algorithm on the continuous data [53] with the assumption that the recorded activity is a linear sum of independent components arising from brain and non-brain, artifact sources. For systematic removal of components representing ocular and cardiac artifacts the EEGLAB-plug-in CORRMAP [54], enabling semi-automatic component identification was used. After artifact attenuation by back-projection of all but the artifactual independent components, the cleaned data was selectively averaged for each condition from the onset of the stimulus, which included 200 ms pre-stimulus baselines and a 600 ms time window. In order to explore differences between non-verbal emotion cue conditions, ERP waveforms and topographical maps for each emotion were inspected and compared for latency and amplitude of peak voltage activity at the onset of the sentence. Visual inspection of average waveforms showed that distribution of ERP effects was predominantly fronto-central. Therefore, peak amplitude and latency analyses were conducted at Cz electrode for each of the selected peaks: N100 as well as P200.

### Statistical analysis

The behavioral as well as ERP measures were subjected to SPSS (10.1) for statistical analysis. The reaction time and accuracy rate were analyzed with 3×2×2 repeated measures analyses of variance (ANOVA), with emotional prosody [neutral, angry, happy] and stimulus type [unsimulated, simulated] as within-subjects factors, whereas strategy [ACE, PACE] served as between-subjects factor. All ERP analysis followed the same ANOVA design as the behavioral analysis. In order to correct for sphericity violation (p < 0.05), the Greenhouse-Geisser correction was used in relevant cases. Significant interactions were followed by paired *t*-test to examine the relationship between emotional prosody, stimulus type and strategy.

## Abbreviations

ERPs: Event related potentials; NH: Normal hearing; CIs: Cochlear implants; ACE: Advanced Combination Encoder; PACE: Psychoacoustic Advanced Combination Encoder; HSM: Hochmair, Schulz, and Moser sentence test; BDI: Becks depression inventory.

## Competing interests

The authors declare that they have no competing interests.

## Authors’ contributions

DA performed the experiment, analyzed the data and drafted the manuscript. LT participated in the design of the study and the collection of data. FCV and SD participated in analysis of the data, and reviewed the manuscript. AB participated in creating the simulations and reviewed the manuscript. RD reviewed the manuscript. MW participated in its design and coordination and helped to draft the manuscript. All authors read and approved the final manuscript.

## References

[B1] Ross ED The aprosodias. Functional-anatomic organization of the affective components of language in the right hemisphere Arch Neurol 1981 38 9 561 569 10.1001/archneur.1981.00510090055006 7271534

[B2] Murray IR Arnott JL Toward the simulation of emotion in synthetic speech: a review of the literature on human vocal emotion J Acoust Soc Am 1993 93 2 1097 1108 10.1121/1.405558 8445120

[B3] Schroder C Mobes J Schutze M Szymanowski F Nager W Bangert M Munte TF Dengler R Perception of emotional speech in Parkinson's disease Mov Disord 2006 21 10 1774 1778 10.1002/mds.21038 16830324

[B4] Nikolova ZT Fellbrich A Born J Dengler R Schroder C Deficient recognition of emotional prosody in primary focal dystonia Eur J Neurol 2011 18 2 329 336 10.1111/j.1468-1331.2010.03144.x 20666836

[B5] Chee GH Goldring JE Shipp DB Ng AH Chen JM Nedzelski JM Benefits of cochlear implantation in early-deafened adults: the Toronto experience J Otolaryngol 2004 33 1 26 31 10.2310/7070.2004.01074 15291273

[B6] Kaplan DM Shipp DB Chen JM Ng AH Nedzelski JM Early-deafened adult cochlear implant users: assessment of outcomes J Otolaryngol 2003 32 4 245 249 10.2310/7070.2003.41601 14587565

[B7] Donaldson GS Nelson DA Place-pitch sensitivity and its relation to consonant recognition by cochlear implant listeners using the MPEAK and SPEAK speech processing strategies J Acoust Soc Am 2000 107 3 1645 1658 10.1121/1.428449 10738818

[B8] Sandmann P Dillier N Eichele T Meyer M Kegel A Pascual-Marqui RD Marcar VL Jancke L Debener S Visual activation of auditory cortex reflects maladaptive plasticity in cochlear implant users Brain 2012 135 Pt 2 555 568 22232592 10.1093/brain/awr329

[B9] Mohr PE Feldman JJ Dunbar JL McConkey-Robbins A Niparko JK Rittenhouse RK Skinner MW The societal costs of severe to profound hearing loss in the United States Int J Technol Assess Health Care 2000 16 4 1120 1135 10.1017/S0266462300103162 11155832

[B10] Shannon RV Zeng FG Kamath V Wygonski J Ekelid M Speech recognition with primarily temporal cues Science 1995 270 5234 303 304 10.1126/science.270.5234.303 7569981

[B11] Buechner A Brendel M Krueger B Frohne-Buchner C Nogueira W Edler B Lenarz T Current steering and results from novel speech coding strategies Otol Neurotol 2008 29 2 203 207 10.1097/mao.0b013e318163746 18223448

[B12] Nogueira W Vanpoucke F Dykmans P De Raeve L Van Hamme H Roelens J Speech recognition technology in CI rehabilitation Cochlear Implants Int 2010 11 Suppl 1 449 453 21756671 10.1179/146701010X12671177204507

[B13] Loizou PC Signal-processing techniques for cochlear implants IEEE Eng Med Biol Mag 1999 18 3 34 46 10.1109/51.765187 10337562

[B14] Nogueira W Buechner A Lenarz T Edler B A Psychoacoustic "NofM"-type speech coding strategy for cochlear implants J Appl Signal Process Spec Issue DSP Hear Aids Cochlear Implants Eurasip 2005 127 18 3044 3059

[B15] Lai WK Dillier N Investigating the MP3000 coding strategy for music perception 11 Jahrestagung der Deutschen Gesellschaft für Audiologie: 2008 2008 Germany: Kiel 1 4

[B16] Weber J Ruehl S Buechner A Evaluation der Sprachverarbeitungsstrategie MP3000 bei Erstanpassung81st Annual Meeting of the German Society of Oto-Rhino-Laryngology, Head and Neck Surgery 2010 Wiesbaden: German Medical Science GMS Publishing House

[B17] Kutas M Hillyard SA Event-related brain potentials to semantically inappropriate and surprisingly large words Biol Psychol 1980 11 2 99 116 10.1016/0301-0511(80)90046-0 7272388

[B18] Steinhauer K Alter K Friederici AD Brain potentials indicate immediate use of prosodic cues in natural speech processing Nat Neurosci 1999 2 2 191 196 10.1038/5757 10195205

[B19] Schapkin SA Gusev AN Kuhl J Categorization of unilaterally presented emotional words: an ERP analysis Acta Neurobiol Exp (Wars) 2000 60 1 17 28 10769926 10.55782/ane-2000-1321

[B20] Kotz SA Meyer M Alter K Besson M von Cramon DY Friederici AD On the lateralization of emotional prosody: an event-related functional MR investigation Brain Lang 2003 86 3 366 376 10.1016/S0093-934X(02)00532-1 12972367

[B21] Pihan H Altenmuller E Ackermann H The cortical processing of perceived emotion: a DC-potential study on affective speech prosody Neuroreport 1997 8 3 623 627 10.1097/00001756-199702100-00009 9106735

[B22] Kotz SA Paulmann S When emotional prosody and semantics dance cheek to cheek: ERP evidence Brain Res 2007 1151 107 118 17445783 10.1016/j.brainres.2007.03.015

[B23] Hillyard SA Picton TW On and off components in the auditory evoked potential Percept Psychophys 1978 24 5 391 398 10.3758/BF03199736 745926

[B24] Rosburg T Boutros NN Ford JM Reduced auditory evoked potential component N100 in schizophrenia–a critical review Psychiatr Res 2008 161 3 259 274 10.1016/j.psychres.2008.03.017 18926573

[B25] Anderson L Shimamura AP Influences of emotion on context memory while viewing film clips Am J Psychol 2005 118 3 323 337 16255123

[B26] Zeelenberg R Wagenmakers EJ Rotteveel M The impact of emotion on perception: bias or enhanced processing? Psychol Sci 2006 17 4 287 291 10.1111/j.1467-9280.2006.01700.x 16623684

[B27] Grandjean D Sander D Pourtois G Schwartz S Seghier ML Scherer KR Vuilleumier P The voices of wrath: brain responses to angry prosody in meaningless speech Nat Neurosci 2005 8 2 145 146 10.1038/nn1392 15665880

[B28] Grandjean D Sander D Lucas N Scherer KR Vuilleumier P Effects of emotional prosody on auditory extinction for voices in patients with spatial neglect Neuropsychologia 2008 46 2 487 496 10.1016/j.neuropsychologia.2007.08.025 17945316

[B29] Scherer KR Vocal communication of emotion: a review of research paradigms Speech Comm 2003 40 227 256 10.1016/S0167-6393(02)00084-5

[B30] Luo X Fu QJ Frequency modulation detection with simultaneous amplitude modulation by cochlear implant users J Acoust Soc Am 2007 122 2 1046 1054 10.1121/1.2751258 17672652

[B31] Seither-Preisler A Patterson R Krumbholz K Seither S Lutkenhoner B Evidence of pitch processing in the N100m component of the auditory evoked field Hear Res 2006 213 1–2 88 98 16464550 10.1016/j.heares.2006.01.003

[B32] Schirmer A Kotz SA Beyond the right hemisphere: brain mechanisms mediating vocal emotional processing Trends Cogn Sci 2006 10 1 24 30 10.1016/j.tics.2005.11.009 16321562

[B33] Pinheiro AP Galdo-Alvarez S Rauber A Sampaio A Niznikiewicz M Goncalves OF Abnormal processing of emotional prosody in Williams syndrome: an event-related potentials study Res Dev Disabil 2011 32 1 133 147 10.1016/j.ridd.2010.09.011 20961731

[B34] Garcia-Larrea L Lukaszevicz AC Mauguiere F Revisiting the oddball paradigm. Non-target vs. neutral stimuli and the evaluation of ERP attentional effects Neuropsychologia 1992 30 723 741 10.1016/0028-3932(92)90042-K 1407488

[B35] Alain C Woods DL Covarrubias D Activation of duration-sensitive auditory cortical fields in humans Electroencephalogr Clin Neurophysiol 1997 104 6 531 539 10.1016/S0168-5597(97)00057-9 9402895

[B36] Picton TW Goodman WS Bryce DP Amplitude of evoked responses to tones of high intensity Acta Otolaryngol 1970 70 2 77 82 10.3109/00016487009181862 5472109

[B37] Meyer M Baumann S Jancke L Electrical brain imaging reveals spatio-temporal dynamics of timbre perception in humans NeuroImage 2006 32 4 1510 1523 10.1016/j.neuroimage.2006.04.193 16798014

[B38] Shahin A Bosnyak DJ Trainor LJ Roberts LE Enhancement of neuroplastic P2 and N1c auditory evoked potentials in musicians J Neurosci 2003 23 13 5545 5552 12843255 10.1523/JNEUROSCI.23-13-05545.2003 PMC6741225

[B39] Paulmann S Pell MD Kotz SA How aging affects the recognition of emotional speech Brain Lang 2008 104 3 262 269 10.1016/j.bandl.2007.03.002 17428529

[B40] Kotz SA Meyer M Paulmann S Lateralization of emotional prosody in the brain: an overview and synopsis on the impact of study design Prog Brain Res 2006 156 285 294 17015086 10.1016/S0079-6123(06)56015-7

[B41] Alter K Rank E Kotz SA Toepel U Besson M Schirmer A Friederici AD Affective encoding in the speech signal and in event-related brain potentials Speech Comm 2003 40 61 70 10.1016/S0167-6393(02)00075-4

[B42] Johnstone T van Reekum CM Oakes TR Davidson RJ The voice of emotion: an FMRI study of neural responses to angry and happy vocal expressions Soc Cogn Affect Neurosci 2006 1 3 242 249 10.1093/scan/nsl027 17607327 PMC1905858

[B43] Spreckelmeyer KN Kutas M Urbach T Altenmuller E Munte TF Neural processing of vocal emotion and identity Brain Cogn 2009 69 1 121 126 10.1016/j.bandc.2008.06.003 18644670 PMC2642974

[B44] Lang SF Nelson CA Collins PF Event-related potentials to emotional and neutral stimuli J Clin Exp Neuropsychol 1990 12 6 946 958 10.1080/01688639008401033 2286657

[B45] Qin MK Oxenham AJ Effects of simulated cochlear-implant processing on speech reception in fluctuating maskers J Acoust Soc Am 2003 114 1 446 454 10.1121/1.1579009 12880055

[B46] Laneau J Wouters J Moonen M Relative contributions of temporal and place pitch cues to fundamental frequency discrimination in cochlear implantees J Acoust Soc Am 2004 116 6 3606 3619 10.1121/1.1823311 15658711

[B47] Drennan WR Rubinstein JT Music perception in cochlear implant users and its relationship with psychophysical capabilities J Rehabil Res Dev 2008 45 5 779 789 10.1682/JRRD.2007.08.0118 18816426 PMC2628814

[B48] Wittfoth M Schroder C Schardt DM Dengler R Heinze HJ Kotz SA On emotional conflict: interference resolution of happy and angry prosody reveals valence-specific effects Cereb Cortex 2010 20 2 383 392 10.1093/cercor/bhp106 19505993

[B49] Swanson B Mauch H Nucleus MATLAB Toolbox Software User Manual 2006

[B50] Boersma P Weenink D Praat: doing phonetics by computer 2005

[B51] Jasper H Progress and problems in brain research J Mt Sinai Hosp N Y 1958 25 3 244 253 13525936

[B52] Delorme A Makeig S EEGLAB: an open source toolbox for analysis of single-trial EEG dynamics including independent component analysis J Neurosci Meth 2004 134 1 9 21 10.1016/j.jneumeth.2003.10.009 15102499

[B53] Debener S Thorne J Schneider TR Viola FC Using ICA for the analysis of multi-channel EEG data Simultaneous EEG and fMRI Edited by Debener MUS 2010 New York, NY: Oxford University Press 121 135

[B54] Viola FC Thorne J Edmonds B Schneider T Eichele T Debener S Semi-automatic identification of independent components representing EEG artifact Clin Neurophysiol 2009 120 5 868 877 10.1016/j.clinph.2009.01.015 19345611

